# Effects of 1-year yoga on cardiovascular risk factors in middle-aged and older adults with metabolic syndrome: a randomized trial

**DOI:** 10.1186/s13098-015-0034-3

**Published:** 2015-04-30

**Authors:** Parco M Siu, Angus P Yu, Iris F Benzie, Jean Woo

**Affiliations:** Department of Health Technology and Informatics, Faculty of Health and Social Sciences, The Hong Kong Polytechnic University, Hung Hom, Hong Kong China; Department of Medicine and Therapeutics, Faculty of Medicine, The Chinese University of Hong Kong, Shatin, Hong Kong China

**Keywords:** Body-mind exercise, Diabetes mellitus, Hypertension, Central obesity

## Abstract

**Background:**

Metabolic syndrome (MetS) is a clustering of cardiovascular risk factors, which is associated with diabetes mellitus and cardiovascular disease. Lifestyle interventions applied to people with MetS has considerable beneficial effects on disease preventive outcomes. This study aimed to examine the effects of 1-year of yoga exercise on the cardiovascular risk factors including central obesity, hypertension, dyslipidemia and hyperglycemia in middle-aged and older Hong Kong Chinese adults with MetS.

**Methods:**

Adults diagnosed with MetS using National Cholesterol Education Program criteria (n = 182; mean ± SD age = 56 ± 9.1) were randomly assigned to a 1-year yoga intervention group or control group. Systolic and diastolic blood pressure, waist circumference, fasting plasma glucose, triglycerides, and high-density lipoprotein cholesterol were examined at baseline, midway, and on completion of the study. Physical activity level and caloric intake were assessed and included in the covariate analyses.

**Results:**

A reduction of the number of diagnostic components for MetS was found to be associated with the yoga intervention. Waist circumference was significantly improved after the 1-year yoga intervention. A trend towards a decrease in systolic blood pressure was observed following yoga intervention.

**Conclusion:**

These results suggest that yoga exercise improves the cardiovascular risk factors including central obesity and blood pressure in middle-aged and older adults with MetS. These findings support the complementary beneficial role of yoga in managing MetS.

## Introduction

Metabolic syndrome (MetS) refers to a clustering of cardiovascular risk factors, including high blood pressure, central obesity, insulin resistance and dyslipidemia, that has become a global public health epidemic. MetS represents a pre-diabetic and pre-cardiovascular pathological condition, and could be taken as a useful tool in prognosing development of cardiovascular disease and diabetes mellitus [1-5]. Most importantly, lifestyle modification interventions applied at the pre-pathologic stage signified by MetS could be of considerable health benefit and clinical significance, achieving better preventive and therapeutic outcomes in regard to diabetes and cardiovascular disease [4,6]. Indeed, lifestyle modification has been suggested to be the cornerstone for successful management of MetS [6,7].

Yoga is a mind-body exercise which comprises meditation, breathing and body posture [8]. Hatha yoga, a branch of yoga that is most widely practiced, consists of manipulation of respiration (pranayama) and posture control (asana). In addition to voluntary active control of abdominal breathing, Hatha yoga intends to develop a stable and effortless body position by coordinating posture which involves balancing and stretching [9]. Indeed, yoga has been shown to decrease stress [10], reduce depression and anxiety, and increase the perceived self-efficacy in healthy individuals [11,12]. In addition to the beneficial psychological effects, yoga has also been demonstrated to be beneficial for modifying the cardiovascular risk factors. A randomized case control study has demonstrated that 3-month of yoga intervention induces significant improvements of MetS parameters including waist circumference, systolic/diastolic blood pressure, fasting blood sugar level, HbA1c, serum triglyceride and high-density lipoprotein-cholesterol in middle-aged adults [13]. Another randomized controlled pilot study has further illustrated the feasibility and acceptability of yoga intervention in sedentary overweight adults aged ~52 years with MetS [14]. Their data indicated that 10-week of yoga intervention increases energy level, tends to lower blood pressure and improve psychological well-being and perceived stress [14]. Nevertheless, we lack objective scientific evidence from randomized trials of adequate sample size and a sufficiently long experimental period to show the beneficial effects of yoga in managing MetS, particularly in the older population. This study therefore aimed to assess the effects of a 1-year yoga programme on MetS diagnostic parameters as the primary outcome measures in middle-aged and older adults who were already diagnosed with MetS. The effects of yoga on depression and perceived quality of life were also examined as the secondary outcome measures. We tested the hypothesis that 1-year yoga intervention improves the MetS diagnostic parameters in middle-aged and older adults with MetS.

## Methods

### Study design, settings and subject recruitment

**Figure 1:**
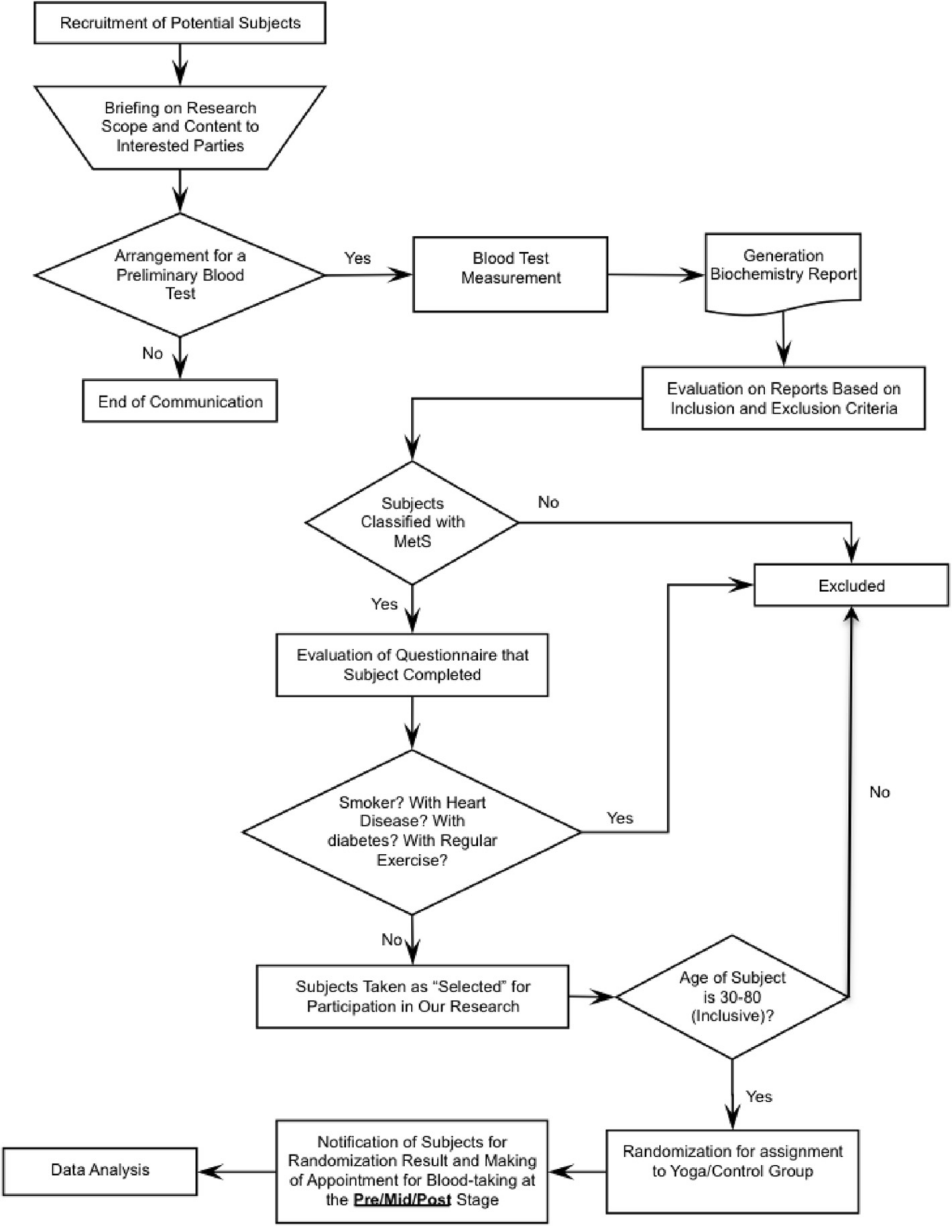
Schematic presentation of the study work flow.

The present study was a randomized controlled, parallel, open label trial in patients with MetS conducted in one center in Hong Kong between November 2010 and Aug 2013. Adults with age between 30 and 80, diagnosed with MetS using National Cholesterol Education Program (NCEP) criteria (n = 182; mean ± SD age = 56 ± 9.1) were randomly assigned to a 1-year yoga intervention group or control group. During the 1-year experimental period, subjects attended three measurement sessions, which were the baseline measurement right after being recruited to the study (Pre), mid-term follow-up at six months after the study started (Mid), and the final measurement upon completion of the 1-year experimental period (Post). The schematic study flowchart is presented in Figure 1. Power analysis was conducted to estimate the sample size needed. With α = 0.05, β = 0.2 and moderate effect size (0.25 standard deviation), total of 86 subjects were needed (43 subjects in each group).

In this study, 3,967 Chinese participants aged from 18 to 94 voluntarily recruited from the community were screened for MetS between 2010 to 2012 on the basis of the diagnostic guideline of the United States National Cholesterol Education Program (NCEP) Expert Panel Adult Treatment Panel (ATP) III criteria, in which an individual diagnosed with MetS has three or more of following characteristics: 1) central obesity (waist circumference exceeds 90 cm or 80 cm for Asian male and female, respectively), 2) hypertension (systolic pressure equals or exceed 130 mmHg or diastolic pressure equals or exceeds 85 mmHg), 3) elevated blood glucose (fasting glucose level equals or exceeds 5.5 mmol/L [100 mg/dL]), 4) elevated plasma triglycerides (level equals or exceeds 1.70 mmol/L [150 mg/dL]), and 5) low level of high-density lipoprotein-cholesterol (HDL-C; level equals or is less than 40 mg/dL for male and 50 mg/dL for female) [5]. Participants were informed about the potential risks and benefits of their participation, and written informed consent was obtained on a voluntary basis before the study began. All the experimental procedures received human research ethics approval from The Hong Kong Polytechnic University (ethics approval number: HSEARS20090820001).

**Figure 2:**
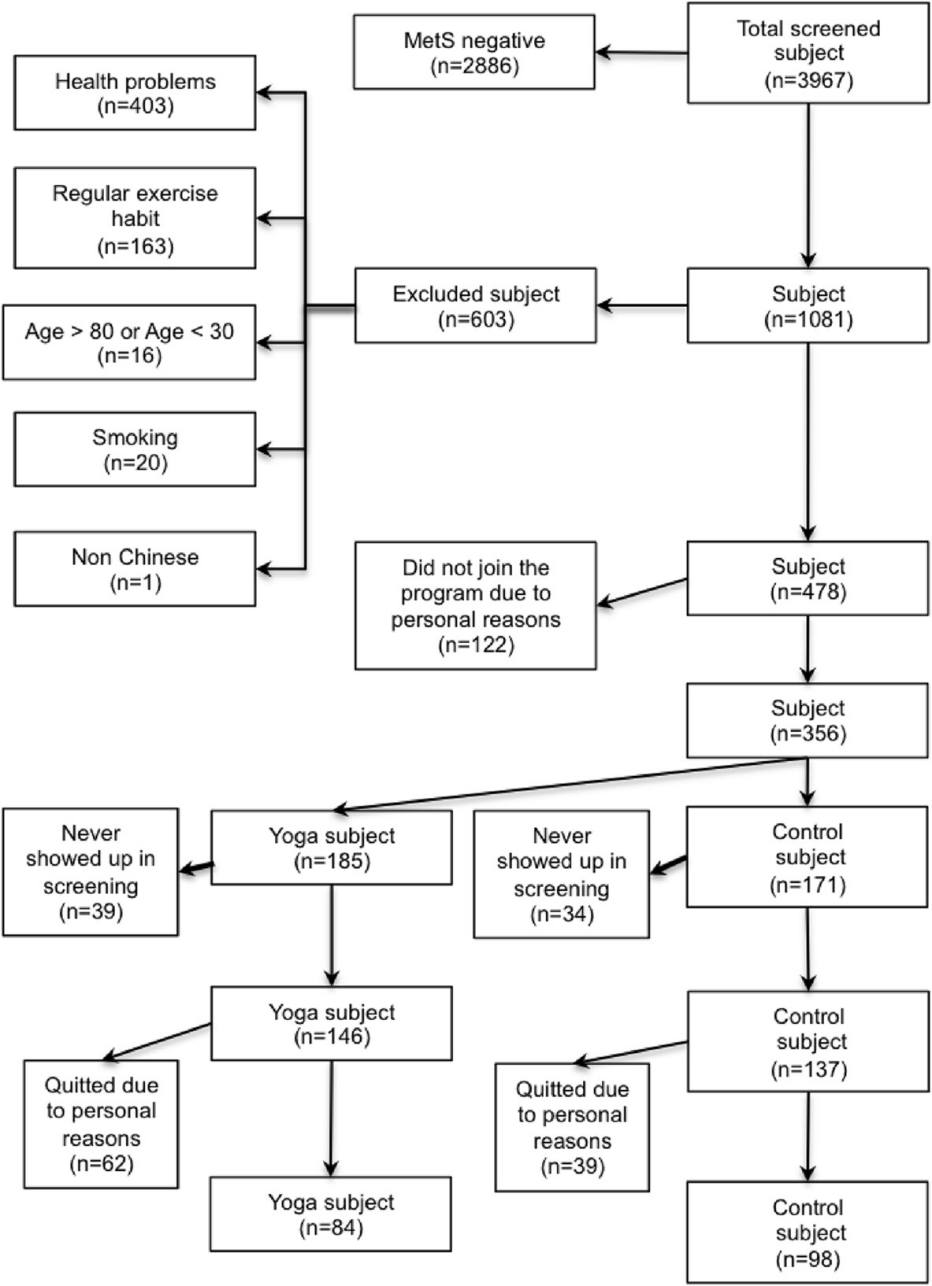
Schematic presentation of the study.

Participants who were not in the age range from 30 to 80 or reported having dementia or mental disorder, severe cardiovascular illness, previous stroke, neuromusculoskeletal illness, diagnosed major orthopaedic problems in the lower back, pelvis or lower extremities, severe rheumatoid arthritis or osteoarthritis, pulmonary illness, or who were immobile or used a wheelchair, on drug therapy treating metabolic abnormalities, had symptomatic heart or lung disease, or other physical conditions not suitable for yoga exercise were excluded. In addition, regular tobacco users and subjects who exercised regularly (three or more days a week in which they had moderate-to-vigorous exercise of duration of at least 30 minutes per session) were excluded. Randomization and allocation to trial group were conducted by a computer program. In this study, 283 adults participants were randomized, and assigned to control group and yoga group (Control Group n = 137, Yoga Group n = 146) and 182 participants finally completed the study (Control Group n = 98, Yoga Group n = 84) (Figure 2).

### Intervention

Among the screened participants, suitable subjects diagnosed with MetS were invited to participate in our yoga intervention study. A computer program which could randomly generate the group names was employed to allocate the suitable participants to control and yoga groups. Subjects in control group were contacted monthly by telephone to assess their health status by questionnaire and monitored throughout the study. Subjects in yoga group were assigned to 1-year of yoga program following a published protocol with customized modifications for yoga beginners and senior individuals [15]. Subjects in the yoga group who failed to achieve 70% attendance to the training program were excluded from the study. All subjects were asked to adhere to their habitual daily dietary intake and other physical activities throughout the experimental period. Subjects received supermarket coupon once they have completed the study as an incentive.

### Yoga intervention

Participants in the yoga group attended three yoga sessions weekly for 1 year. Each session lasted for 60-min consisting of 10-min of warm-up, 40-min of Hatha yoga practice, and 10-min of breathing exercise and relaxation. The postures in the routine training protocol including sukhasana (easy pose), child pose, cat and cow stretch, adho muha svanasana (downward dog), high lunge, uttanasana (standing forward bend), mountain pose, uradva hastasana (upward salute), ukatassanna (chair pose), virabhadrasana (worrior pose), utthita parsva konasana (side angle pose), utthita trikonasana (extended triangle pose), vrksasana (tree pose), malasana (garland pose), eka pada rajakapotasana (one legged king pigeon pose), salambhasana (locust pose), dandasana (staff pose), baddha konasana (bound angle pose), agnistambhasana (fire log pose), gomukhasana (cow face pose), spinal twist, paripurna navasana (knees bend version of boat pose), setu bandhasana (bridge pose), supta padangusthasana (reclining big toe pose), ananda balsana (happy baby pose), centering (in cross-legged position), finger and toe weaving, virasana (hero pose), vajrasana (thunderbolt pose), tadasana (mountain pose), table pose (e.g., leg lifts), salabasana (locust pose), padangusthasana (big toe pose), sputa padangusthana (big toe lying down pose), uttitha hasta padangustasana (extended big toe pose), sputa baddha konasana (lying down bound angle pose), eka pada bhekasana (1-leg frog pose), and shavasana (corpse pose). All these postures have been reported to be suitable for yoga beginners and senior individuals [15]. Subjects were encouraged, but not required, to complete all the tasks. All yoga classes were operated by certified yoga instructors who had at least 5 years of yoga teaching experience. The intensity of yoga exercise was determined by the instructors in order to provide a progressive level of challenge to the subjects. The performance of the subject was continuously and closely monitored by the attending yoga instructor. Adjustment of the intensity of yoga exercise was made according to the judgement of instructor in order to maintain the appropriate challenge for the subjects.

### Outcomes measurements: MetS parameters and blood biochemistry

In each measurement session, subjects of yoga and control groups were assessed for the NCEP-defined MetS diagnostic parameters (including blood pressure, waist circumference, fasting glucose, triglycerides, and HDL-C) and were requested to complete a battery of questionnaires including Center for Epidemiologic Studies Depression Scale (CES-D), the 12-item Short-form health survey (SF-12), and the International Physical Activity Questionnaire (IPAQ). At the Pre and Post measurement sessions, subjects were also requested to provide information of a 3-day weighed dietary record which included two weekdays plus one day during the weekend.

Cardiovascular measurements including systolic blood pressure, diastolic blood pressure, and heart rate were examined by an electronic blood pressure monitor (Accutorr Plus, Datascope). Blood pressure measurement was determined on the right arm after 5-min seated rest. Systolic and diastolic blood pressure were obtained over the brachial artery region with the arm supported at heart level using appropriate sized cuff. The average of two measurements taken with a 1-min interval between them was recorded for analysis. Waist circumference was measured midway between the lowest rib and the superior border of the iliac crest using an inelastic measuring tape on the bare skin and recorded to the nearest 0.1 cm. The tape was snugged horizontally around the abdomen passing across the navel without causing compression on the skin. Measurement was performed at the end of normal expiration. Measurements of blood pressure and waist circumference were conducted by a trained research personnel. Biochemical measurements were performed on fasting plasma harvested from venous blood samples collected after an overnight (a minimum of 10 hours) fast by certified phlebotomists. Plasma glucose, triglycerides, and HDL-cholesterol concentrations were measured by an accredited medical laboratory by commercial test kit methods using an automatic clinical chemistry analyzer (Architect CI8200, Abbott Diagnostics).

### Data analyses

Data are expressed as mean ± standard deviation. Chi-square test was performed to examine categorical data. Mixed design repeated measure analysis of co-variance (ANCOVA) with a 2 × 3 (yoga intervention × time) model was used to analyze the quantitative data. The changes of measured parameters at Mid and Post relative to Pre in control and yoga groups were analyzed by independent t-test. Both per protocol analysis (PP) and intention-to-treat analysis (ITT) were performed. Intention-to-treat analysis was conducted by following a publish protocol with multiple imputation procedure [16]. All statistical analyses were performed using the Statistical Package for the Social Sciences (SPSS) version 21 for Windows. Statistical significance was accepted at *p* < 0.05.

## Results

A total of 3,967 volunteers were screened for metabolic syndrome (MetS), in which MetS was diagnosed in 1,081 subjects (27.2%). Central obesity and high systolic blood pressure were found to be the most common MetS components, being present in ~50% of the screened participants (Table 1). High diastolic blood pressure, elevated plasma glucose and triglycerides, and low HDL-C were found in 22-24% of the screened participants (Table 1).

**Table 1:** **Summary of the frequency and distribution of diagnostic components for MetS in the 3,967 community-based participants screened**

	**Participants screened (n = 3,967)**	
**Age**	55.3 ± 10.7 (range 18 to 94) years	
**Gender**	957 male & 3,010 female	
**No. of subjects had 0 MetS component**	891 (22.5%)	MetS-negative
**No. of subjects had 1 MetS component**	1,084 (27.3%)	
**No. of subjects had 2 MetS components**	911 (23.0%)	
**No. of subjects had 3 MetS components**	627 (15.8%)	MetS-positive
**No. of subjects had 4 MetS components**	355 (8.9%)	
**No. of subjects had 5 MetS components**	99 (2.5%)	
**No. of subjects had central obesity**	1,884 (47.5%)	
**No. of subjects had high systolic blood pressure**	1,974 (49.8%)	
**No. of subjects had high diastolic blood pressure**	883 (22.3%)	
**No. of subjects had elevated plasma glucose**	885 (22.3%)	
**No. of subjects had elevated plasma triglyceride**	934 (23.5%)	
**No. of subjects had reduced HDL-cholesterol**	947 (23.9%)	

According to the diagnostic guideline of NCEP ATP III criteria, an individual is diagnosed with MetS when acquiring three or more of following conditions: 1) central obesity (waist circumference exceeds 90 cm or 80 cm for Asian male and female, respectively), 2) hypertension (systolic pressure equals to or is greater than 130 mmHg or diastolic pressure equals to or is greater than 85 mmHg), 3) elevated blood glucose (fasting plasma glucose level equals to or is higher than 100 mg/dL), 4) elevated blood triglyceride (blood triglyceride level equals to or exceeds 150 mg/dL), and 5) low level of high-density lipoprotein-cholesterol (HDL-C; level equals or is less than 40 mg/dL for male and 50 mg/dL for female). Notably, the numbers of subjects had central obesity, high systolic and diastolic blood pressure, elevated blood glucose, elevated triglyceride, and reduced HDL-cholesterol do not add up to the total number of participants screened (i.e., 3,967) and 100% because subjects had multiple MetS components.

After evaluation of subject’s information for inclusion and exclusion criteria, 182 invited subjects successfully completed the 1-year study (n = 84 in yoga group and n = 98 in the control group) (Figure 2). No significant difference was found in the baseline subject characteristics between yoga and control groups (Table 2). Our Chi-square analysis demonstrated that the change of the number of MetS components after the 1-year experimental period was significantly associated with the yoga intervention (P = 0.026) (Table 3). Indeed, 44% (37/84) of the subjects no longer met the MetS diagnostic threshold at the end of the 1-year yoga intervention (Table 3). In yoga group, 67% of the subjects (56/84) had decreased number of MetS components whereas 15% (13/84) of the subjects had increased number of MetS components after the 1-year experimental period (Table 3). Similar significant results are obtained by using the intention-to-treat analysis (P = 0.02).

**Table 2:** **Baseline comparison of subject characteristics in Control and Yoga groups**

	**Control group (n = 98)**	**Yoga group (n = 84)**	**P-value**
Age	55.7 ± 9.4	56.3 ± 8.8	0.368
Gender	23 male, 75 female	24 male, 60 female	0.433
Waist circumference (cm)	89.2 ± 6.3	89.9 ± 7.7	0.523
Systolic blood pressure (mmHg)	132.8 ± 17.8	135.0 ± 15.5	0.376
Diastolic blood pressure (mmHg)	81.3 ± 10.5	83.6 ± 9.0	0.121
Fasting plasma glucose (mmol/L)	5.8 ± 1.0	5.7 ± 1.3	0.164
Fasting plasma triglycerides (mmol/L)	2.4 ± 2.2	2.0 ± 1.0	0.164
Fasting plasma HDL-C (mmol/L)	1.2 ± 0.3	1.2 ± 0.2	0.742
Resting heart rate (beat/min)	71.0 ± 8.6	71.2 ± 10.0	0.792
IPAQ activity (MET-min/week)	2857.6 ± 4953.4	3629.8 ± 2735.7	0.444
IPAQ sitting (min/week)	2291.6 ± 1494.5	2485.2 ± 1563.3	0.973
Average daily calories intake (kcal)	2313.0 ± 984.4	2293.6 ± 815.8	0.922

**Table 3:** **Summary of the frequency and distribution of diagnostic components for MetS at baseline (Pre), midway (Mid), and completion (Post) of the 1-year experimental period in Control and Yoga groups**

	**Control group (n=98 for PP, n=137 for ITT)**			**Yoga group (n=84 for PP, n=146 for ITT)**			**P-value**	**Effect size**	**P-value**	**Effect size**
**Age**	55.7 ± 9.4 (range 37 to 77) years			56.3 ± 8.8 (range 38 to 75) years			(PP)	(PP)	(ITT)	(ITT)
**No. of subjects recovered from MetS after 1-yr (i.e., < 3 components)**	34			37			0.197	0.061	0.150	0.064
**No. of subjects had increased no. of MetS components after 1-yr**	13			13			0.026*	0.146	0.020*	0.134
**No. of subjects had unchanged no. of MetS component after 1-yr**	35			15						
**No. of subjects had decreased no. of MetS components after 1-yr**	50			56						
	**Pre**	**Mid**	**Post**	**Pre**	**Mid**	**Post**				
**No. of subjects had 0 MetS component**	0 (0%)	1 (1.0%)	4 (4.1%)	0 (0%)	1 (1.2%)	4 (4.8%)	-	-	-	-
**No. of subjects had 1 MetS component**	0 (0%)	8 (8.2%)	9 (9.2%)	0 (0%)	10 (11.9%)	11 (13.1%)	-	-	-	-
**No. of subjects had 2 MetS components**	0 (0%)	25 (25.5%)	21 (21.4%)	0 (0%)	16 (19.1%)	22 (26.2%)	-	-	-	-
**No. of subjects had 3 MetS components**	55 (56.1%)	26 (26.5%)	32 (32.6%)	50 (59.5%)	35 (41.7%)	26 (31.0%)	-	-	-	-
**No. of subjects had 4 MetS components**	32 (32.6%)	30 (30.6%)	25 (25.5%)	26 (31.0%)	21 (25.0%)	20 (23.8%)	-	-	-	-
**No. of subjects had 5 MetS components**	11 (11.2%)	8 (8.2%)	7 (7.1%)	8 (9.5%)	1 (1.2%)	1 (1.2%)	-	-	-	-
**No. of subjects had central obesity**	93 (94.9%)	77 (78.6%)	80 (81.6%)	76 (90.5%)	66 (78.6%)	54 (64.3%)	0.018**	0.145	0.279	0.378
**No. of subjects had high systolic blood pressure**	61 (62.2%)	54 (55.1%)	49 (50.0%)	52 (61.9%)	43 (51.2%)	40 (47.6%)	0.497	0.045	0.463	0.358
**No. of subjects had high diastolic blood pressure**	41 (41.8%)	36 (36.7%)	33 (33.7%)	39 (46.4%)	25 (29.8%)	27 (32.1%)	0.428	0.063	0.491	0.346
**No. of subjects had elevated fasting plasma glucose**	48 (49.0%)	42 (42.9%)	34 (34.7%)	43 (51.2%)	38 (45.2%)	32 (38.1%)	0.644	0.034	0.512	0.359
**No. of subjects had elevated fasting plasma triglycerides**	72 (73.5%)	60 (61.2%)	60 (61.2%)	52 (61.9%)	48 (57.1%)	42 (50.0%)	0.758	0.019	0.372	0.362
**No. of subjects had low plasma HDL-cholesterol**	67 (68.4%)	55 (56.1%)	55 (56.1%)	57 (67.9%)	46 (54.8%)	44 (52.4%)	0.580	0.035	0.479	0.350

According to the diagnostic guideline of NCEP ATP III criteria, an individual is diagnosed with MetS when acquiring three or more of following conditions: 1) central obesity (waist circumference exceeds 90 cm or 80 cm for Asian male and female, respectively), 2) hypertension (systolic pressure equals to or is greater than 130 mmHg or diastolic pressure equals to or is greater than 85 mmHg), 3) elevated blood glucose (fasting plasma glucose level equals to or is higher than 100 mg/dL), 4) elevated blood triglyceride (blood triglyceride level equals to or exceeds 150 mg/dL), and 5) low level of high-density lipoprotein-cholesterol (HDL-C; level equals or is less than 40 mg/dL for male and 50 mg/dL for female). *Change of no. of MetS components after 1-yr experimental period (increased, unchanged or decreased) was significantly associated with the intervention (yoga or control), **Diagnostic change of the presence of central obesity after 1-yr experimental period (still fulfilled the MetS criterion as central obesity or no longer met with the MetS criterion as central obesity) was significantly associated with the intervention (yoga or control) according to our Chi-square analysis. PP; per protocol analysis, ITT; intention-to-treat analysis.

We further examined the change in each individual component to assess if it improved to the extent that it no longer met the MetS defining criterion after the 1-year intervention. As can be seen in Table 3, improvements were seen in all MetS components, but only the decrease in prevalence of central obesity (90.5% to 64.3% at the end of the intervention) reached statistical significance (P = 0.018). However, this change was not found to reach the statistical significance under the intention-to-treat analysis.

According to our mixed design repeated measures ANCOVA analysis, yoga intervention was found to significantly reduce the waist circumference (interaction effect: yoga intervention × time, P = 0.003) which was co-varied with the IPAQ activity index (interaction effect: Change in IPAQ activity × time, P = 0.024) (Table 4). The results of intention-to-treat analysis led to similar conclusions (interaction effect: yoga intervention × time, P = 0.041, interaction effect: Change in IPAQ activity × time, P = 0.001). A trend was observed for yoga intervention to decrease the systolic blood pressure (interaction effect: yoga intervention × time, P = 0.067) in per protocol analysis but not in intention-to-treat analysis (Table 4). Our analysis revealed that yoga intervention significantly decreased the resting heart rate (interaction effect: yoga intervention × time, P = 0.039) which was co-varied with the IPAQ activity index (interaction effect: Change in IPAQ activity × time, P = 0.001) (Table 4), however, the significant change in resting heart rate was not found in the analysis of intention-to-treat. No significant changes of depression level and quality of life index were observed (Table 4). The summarized changes of measured parameters at 6-months (Mid) and 12-months (Post) relative to baseline (Pre) in yoga and control groups were shown in Table 5. No significant changes in the dietary pattern on carbohydrate, protein, dietary fiber, soluble fiber, sugar, fat, and cholesterol at baseline and completion of the 1-year experimental period were observed in yoga and control groups (Table 6).

**Table 4:** **Summary of the changes of waist circumference, systolic and diastolic blood pressure, fasting plasma glucose, triglycerides, high density lipoprotein cholesterol (HDL-C), heart rate, and International Physical Activity Question (IPAQ) activity and sitting levels at baseline (Pre), midway (Mid), and completion (Post) of the 1-year experimental period in Control and Yoga groups**

	**Control group (n=98 for PP, n=137 for ITT)**			**Yoga group (n=84 for PP, n=146 for ITT)**						
	**Pre**	**Mid**	**Post**	**Pre**	**Mid**	**Post**	**P-value (PP)**	**Effect size (PP)**	**P-value (ITT)**	**Effect size (ITT)**
**Waist circumference (cm)**	89.2 ± 6.3	87.4 ± 7.0	89.5 ± 7.6	89.9 ± 7.7	87.78 ± 8.3	86.8 ± 6.9	0.003^#^	0.010	0.041^#^	0.005
							0.024^##^	0.027	0.001^##^	0.019
**Systolic pressure (mmHg)**	132.8 ± 17.8	131.5 ± 17.0	130.2 ± 18.1	135.0 ± 15.5	129.1 ± 13.8	128.5 ± 13.8	0.067	0.021	0.001^##^	0.019
**Diastolic pressure (mmHg)**	81.3 ± 10.5	80.4 ± 9.8	79.7 ± 10.1	83.6 ± 9.0	79.8 ± 9.6	80.0 ± 10.4	0.107	0.019	0.001^###^	0.012
**Fasting plasma glucose (mmol/L)**	5.8 ± 1.0	5.6 ± 0.9	5.6 ± 1.2	5.7 ± 1.3	5.6 ± 1.4	5.5 ± 1.4	0.567	0.004	0.026^###^	0.002
**Fasting plasma triglycerides (mmol/L)**	2.4 ± 2.2	2.3 ± 2.1	2.1 ± 1.2	2.0 ± 1.0	2.0 ± 1.3	1.8 ± 0.7	0.891	0.007	0.007^###^	0.009
**Fasting plasma HDL-C (mmol/L)**	1.2 ± 0.3	1.2 ± 0.3	1.2 ± 0.3	1.2 ± 0.2	1.2 ± 0.2	1.2 ± 0.2	0.567	0.003	0.031^###^	0.003
**Resting heart rate (beats/min)**	71.0 ± 8.6	71.3 ± 10.8	71.0 ± 10.7	71.2 ± 10.0	70.9 ± 8.3	67.8 ± 7.5	0.039^#^	0.006	0.016^###^	0.003
							0.001^##^	0.011		0.005
**IPAQ activity (MET-minutes/week)**	2857.6 ± 4953.4	2607.8 ± 2064.3	3386.6 ± 3745.9	3629.8 ± 2735.7	3608.9 ± 4223.0	3977.7 ± 3946.4	0.051	0.009	0.001^###^	0.021
**IPAQ sitting (minutes/week)**	2291.6 ± 1494.5	2101.1 ± 1479.7	1973.0 ± 1517.8	2485.2 ± 1563.3	2256.1 ± 1223.6	2233.3 ± 1274.7	0.004^###^	0.014	0.044^###^	0.009
**SF-12-PCS**	46.64 ± 6.99	47.01 ± 7.98	47.00 ± 8.21	44.06 ± 8.02	47.21 ± 7.54	47.20 ± 7.94	0.031^###^	0.010	0.039^###^	0.015
**SF-12-MCS**	52.30 ± 8.16	51.21 ± 8.30	52.09 ±6.80	50.58 ± 8.84	51.01 ± 8.97	51.76 ±8.70	0.268	0.003	0.073	0.005
**CES-D**	11.62 ± 7.23	11.77 ± 6.58	10.47 ± 6.64	12.58 ± 7.86	11.85 ± 7.37	12.38 ± 8.53	0.179	0.001	0.631	0.001

^#^Significant interaction effect existed between yoga intervention and time, ^##^Significant interaction effect existed between the change in IPAQ activity and time according to our mixed design repeated measure analysis of co-variance (ANCOVA) with a 2 × 3 (Change in IPAQ × time) model. ^###^Significant main effect of time existed according to our mixed design repeated measure analysis of variance (ANOVA) with a 2 × 3 (yoga intervention × time) model. PP; per protocol analysis, ITT; intention-to-treat analysis.

**Table 5:** **Summary of the changes in waist circumference, systolic and diastolic blood pressure, fasting plasma glucose, triglycerides, HDL-C, resting heart rate, IPAQ activity and sitting indices, and average daily calories intake at Mid and Post when compared to Pre in Control and Yoga groups**

	**Mid compared to Pre**						**Post compared to Pre**					
	**Control group**	**Yoga group**	**P-value (PP)**	**Effect size (PP)**	**P-value (ITT)**	**Effect size (PP)**	**Control group**	**Yoga group**	**P-value (PP)**	**Effect size (PP)**	**P-value (ITT)**	**Effect size (ITT)**
**Change in waist circumference (cm)**	-1.78 ± 5.54	-2.09 ± 5.11	0.693	0.152	0.364	0.152	+0.37 ± 9.50	-3.07 ± 6.12	0.005*	0.444	0.022*	0.326
**Change in systolic blood pressure (mmHg)**	-1.27 ± 13.49	-5.90 ± 13.73	0.023*	0.230	0..08	0.241	-2.63 ± 16.82	-6.50 ± 14.36	0.100	0.235	0.145	0.208
**Change in diastolic blood pressure (mmHg)**	-1.76 ± 12.67	-3.82 ± 9.30	0.218	0.290	0.765	0.261	-1.60 ± 9.91	-3.58 ± 10.51	0.193	0.180	0.242	0.172
**Change in fasting plasma glucose (mmol/L)**	-0.10 ± 0.72	-0.16 ± 0.57	0.551	0.066	0.724	0.057	-0.14 ± 0.98	-0.09 ± 0.61	0.659	0.065	0.515	0.097
**Change in fasting plasma triglycerides (mmol/L)**	-0.08 ± 1.40	-0.02 ± 0.83	0.744	0.045	0.744	0.044	-0.28 ± 1.47	-0.21 ± 0.78	0.703	0.046	0.692	0.059
**Change in fasting plasma HDL-C (mmol/L)**	+0.02 ± 0.22	+0.01 ± 0.16	0.532	0.085	0.537	0.090	+0.06 ± 0.30	+0.03± 0.15	0.338	0.128	0.495	0.094
**Change in resting heart rate (beat/min)**	+0.44 ± 10.31	-0.57 ± 8.00	0.466	0.006	0.648	0.274	-0.02 ± 10.09	-3.52 ± 6.72	0.006*	0.057	0.698	0.218
**Change in IPAQ activity (MET-minutes/week)**	-566.18 ± 5037.04	+98.71 ± 2203.14	0.264	0.252	0.476	0.127	+358.13 ±3598.22	+457.72 ± 2840.62	0.838	0.325	0.152	0.240
**Change in IPAQ sitting (minutes/week)**	-284.64 ± 1318.65	-255.51 ± 1002.39	0.869	0.010	0.845	0.029	-347.86 ± 1560.47	-199.76 ± 1192.26	0.479	0.318	0.126	0.251
**SF-12-PCS**	+0.61 ± 8.41	+3.41 ± 8.00	0.237	0.200	0.234	0.191	+1.72 ± 8.11	+3.41 ± 8.82	0.032*	0.341	0.044*	0.295
**SF-12-MCS**	-1.67 ± 8.21	-0.49 ± 7.91	0.338	0.149	0.606	0.076	-0.78 ±7.41	-0.29 ± 7.42	0.685	0.068	0.504	0.063
**CES-D**	+0.45 ± 6.75	-0.93 ± 6.17	0.182	0.214	0.385	0.129	-1.46 ± 7.73	-0.75 ± 6.88	0.555	0.098	0.511	0.102
**Change in average daily calories intake (kcal)**	N/A	N/A	N/A	N/A	N/A	N/A	-126.45 ± 559.36	63.78 ± 879.94	0.244	0.246	0.244	0.246

*Significant difference existed in the changes between Yoga and Control groups. PP; per protocol analysis, ITT; intention-to-treat analysis.

**Table 6:** **Dietary pattern at baseline (Pre) and completion (Post) of the 1-year experimental period in Control and Yoga groups**

	**Control group (n = 40)**		**Yoga group (n = 40)**			
	**Pre**	**Post**	**Pre**	**Post**	**P-value (PP)**	**Effect size (PP)**
**Carbohydrate (g)**	341.28 ± 147.59	396.57 ± 426.49	361.66 ± 148.16	363.00 ± 176.71	0.403	0.003
**Protein (g)**	104.16 ± 45.31	109.33 ± 75.82	117.78 ± 61.56	108.51 ± 45.39	0.785	0.001
**Dietary fiber (g)**	20.07 ± 9.13	22.24 ± 18.22	29.18 ± 54.19	20.78 ± 13.45	0.482	0.003
**Soluble fiber (g)**	1.85 ± 2.12	1.69 ± 2.01	3.30 ± 5.82	2.67 ± 2.89	0.423	0.003
**Sugar (g)**	57.65 ± 38.72	60.56 ± 54.65	61.65 ± 47.47	56.26 ± 62.42	0.891	0.001
**Fat (g)**	60.55 ± 71.20	61.15 ± 72.99	74.26 ± 117.91	49.92 ± 21.46	0.834	0.006
**Cholesterol (g)**	0.35 ± 0.26	0.29 ± 0.19	0.43 ± 0.50	0.34 ± 0.23	0.095	0.012

## Discussion

Metabolic syndrome (MetS) is generally diagnosed by the presence of characteristics that include abdominal obesity, high blood pressure, hyperglycemia, hypertriglyceridemia, and low high-density lipoprotein (HDL)-cholesterol, with thresholds based on the definitions of different professional bodies such as National Cholesterol Education Program (NCEP) and International Diabetes Federation (IDF) [4,17]. Prevalence of MetS had been continuously increasing over the past decades and has reached epidemic proportions. In the United States, prevalence of MetS in adults increased from 23.7% in 1988–1994 [18] to 34.5% in 1999–2002 [19] based on the data of National Health and Nutrition Examination Survey (NHANES). In Hong Kong, the prevalence of MetS has also increased, from 9% in the early 1990s (based on data obtained from 1,513 Chinese aged 18–66 years) to 23% in 2001–2002 (based on data obtained from 7,473 community-based Chinese aged 19–93 years) [20,21]. In the present study, 3,967 community-based Chinese adults aged 18–94 years were screened for MetS between 2010 and 2012. Our results demonstrated that the prevalence of MetS has further increased to 27% (notably all the aforementioned MetS percentages are defined using the same set of diagnostic criteria, the NCEP criteria, for valid comparison). These findings indisputably illustrate the growing and alarming prevalence of MetS in Hong Kong, which poses a serious public health concern due to the strong link between MetS, diabetes and cardiovascular disease. According to the International Diabetes Federation (IDF), people with MetS have a five-fold greater risk of developing type 2 diabetes and are three times as likely to have a heart attack or stroke relative to people without MetS.

Taking the close relationship between MetS and diabetes as an example, the seriousness and importance of the health problem resulting from MetS was highlighted by Laaksonen and colleagues, who demonstrated that the presence of MetS identified people at high risk for developing diabetes, with odds ratios of 5.0 to 8.8 during a 4-year follow-up in 1,005 Finnish middle-aged men [22]. In the San Antonio Heart Study, MetS predicted the incident rate of diabetes (11.3%) independently of other risk factors such as fasting insulin and impaired glucose tolerance during a 7- to 8-year follow-up in 1,734 participants [23]. Consistent with this, Cheung and colleagues reported that 7.1% of subjects developed diabetes after a 6-year follow-up in 1,679 Hong Kong Chinese adults, and intriguingly these cases of incident diabetes were found to be predicted by MetS at baseline [24]. With the illustrated predictive strength of MetS for diabetic occurrence, MetS can be taken as a warning signal for identifying individuals who are apparently healthy but are susceptible to developing diabetes [24,25]. Our finding of a MetS prevalence of 27% make it clear that MetS has already become an epidemic in Hong Kong, and will lead to increasing rates of diabetes in the community, with serious consequences at the personal, community and socioeconomic levels.

Prevention is the key for restraining the expanding prevalence of MetS, and in those already with MetS. Timely intervention can delay or arrest the progress to the pathologic stage of diabetes and cardiovascular diseases. More cost-effective and acceptable therapeutic tools are needed to lessen the MetS epidemic. Among the currently existing interventions, modification of lifestyle remains the cornerstone for successful management of MetS [6,7]. In regard to lifestyle modification, regular physical activity has been indicated as one of the crucial strategies [26]. Nonetheless, middle-aged and older adult population, who are the age group with the highest prevalence of MetS [18-21], may be unable to adapt or be willing to participate in conventional physical activities, such as running/jogging and gym-based resistance training, but preferring other exercise modalities that are less intense, flexible, and require no special equipment. Yoga offers a suitable exercise option, and in this 1-year intervention study, yoga was found to improve several components of the MetS. According to our analyses, both per protocol analysis and intention-to-treat analysis have indicated that the reduction of the number of diagnostic components for MetS was found to be significantly associated with yoga intervention. Waist circumference was significantly lower after the 1-year yoga intervention according to both analyses of per protocol and intention-to-treat. The systolic blood pressure decreased, though the change did not reach statistical significance under per protocol analysis. These results are consistent with the findings of a recent systematic review and meta-analysis that included 44 randomized controlled trials with a total of 3,168 subjects exhibiting the clinically important effects of yoga on most biological cardiovascular disease risk factors [27].

Central obesity has been implicated as one of the most important risk factors in the development of various chronic diseases including diabetes mellitus and cardiovascular diseases. Recently, there has been increasing data suggesting the favorable effects of yoga exercise training on body weight and body composition (e.g., body fat percentage). Seo and co-workers reported that 8-weeks of yoga training intervention (performed three times per week at intensity of 40-60% of heart rate reserve) significantly reduced body weight, body mass index, fat mass and body fat percentage in obese boys (with body mass index greater than the 95^th^ percentile) aged 14.7 ± 0.5 years (n = 10) when compared to sedentary obese boys in control group aged 14.6 ± 1.0 years (n = 10) [28]. In addition, the observation that regular yoga training improved the body weight and body composition was also subsequently shown in obese postmenopausal women and overweight/obese breast cancer survivors aged between 21–75 years [29,30]. Lee and colleagues demonstrated that body weight, body mass index, lean body mass, body fat percentage, visceral fat area, and waist circumference were significantly decreased in healthy postmenopausal women aged 54.5 ± 2.8 years with more than 36% body fat (N = 16) after a 16-week yoga intervention [29]. Littman and colleagues have also shown that waist circumference was reduced relatively more in women who were overweight/obese (body mass index over 24 kg/m^2^) and breast cancer survivors (n = 63) after 6-months of yoga intervention with 5 yoga practice sessions per week when compared to control subjects [30]. Our present findings of the effects of 1-year of regular yoga training on the improvement of waist circumference have extended the evidence that yoga has favorable effects on body composition in middle-aged and older adults with MetS. The positive effects of yoga intervention clinical trials on weight-related outcome measures such as body mass index, body weight, percent body fat, fat mass, waist circumference, hip circumference, waist-to-hip ratio, and lean mass have recently been reviewed [31]. Yoga exercise training is concluded to be an appropriate and potentially successful lifestyle intervention for weight maintenance, prevention of obesity, and risk reduction for obesity-associated diseases, findings which are further supported by the data presented here and obtained from a reasonably large sample size of middle-aged and older people with MetS and a long experimental study period (i.e., 1-year).

Our observations in regard to the beneficial effects of yoga on blood pressure are in accordance with the previous findings. Singh and coworkers demonstrated significant decreases in systolic and diastolic blood pressure (systolic blood pressure decreased from 142 ± 3.9 to 126 ± 3.2 mmHg; diastolic blood pressure decreased from 86.7 ± 2.5 to 75.5 ± 2.1 mmHg) in 24 type 2 diabetic patients after 40-days of yoga intervention under guidance lasting for 30–40 minutes per day [32]. Cohen and colleagues have shown a trend (P = 0.07) towards reduction of systolic blood pressure in underactive, overweight adult men and women with MetS following 10-weeks of regular yoga training intervention (systolic blood pressure change through the 10-week study period: −3.6 ± 13.9 mmHg in yoga group vs. +5.6 ± 9.3 mmHg in control group, n = 12 in each group) [14]. Besides, a recent randomized controlled trial demonstrated a significant improvement of systolic blood pressure in women aged 30–60 years with MetS following 12-weeks of yoga exercise (systolic blood pressure change through the 12-week study period: −3.5 ± 13.7 mmHg in yoga group, n = 20 *vs.* +2.0 ± 14.7 mmHg in control group, n = 21) [28]. Furthermore, a recent systematic review concluded that yoga is an effective adjunct therapy for hypertension based on the published studies involving a total of 6,693 subjects [29]. In the present study, our results support the use of yoga to lower systolic blood pressure in middle-aged and older people with MetS. While the change was modest (average decrease of 6.5 mmHg), even small decreases in blood pressure have a significant effect on risk of cardiovascular disease and stroke. Notably, the decreasing trend of systolic blood pressure was only observed in per protocol analysis but not intention-to-treat analysis, suggesting that the effects of yoga exercise on systolic blood pressure might depend on the dosage of the intervention and/or subject compliance to the intervention. A control trial in 2014 has demonstrated that the compliance can affect the beneficial effects of yoga intervention on systolic blood pressure. In this study subjects diagnosed with hypertension were assigned to yoga class group (12-week of yoga intervention with one 60-min session per week with advance yoga technique and encourage to perform a 30-min yoga exercise at home every day) and yoga home group (no yoga class but encourage to perform a 15-min yoga exercise at home every day). Intriguingly, the decrease in systolic pressure was only observed in the yoga home group but not in the yoga class group. The researchers suspected that it was because the participants in yoga class group performed fewer yoga sessions compared to the participants in the yoga home group due to barriers including the advanced exercises and the traveling to the class center, causing lower compliance in the yoga class group [33].

In conclusion, the reduction of the number of diagnostic components for MetS was found to be significantly associated with yoga intervention. Waist circumference was improved after the 1-year yoga intervention, while systolic blood pressure tended to be improved following yoga intervention. These results support the beneficial effects of yoga training on the cardiovascular risk factor diagnostic parameters of MetS in middle-aged and older adults. These findings provide scientific evidence on the therapeutic role of yoga in managing MetS. As yoga exercise is an economical regimen which can be easily and readily applied to a large scale of target population, it is of practical usefulness to relieve the burden of cardiovascular disease and diabetes by alleviating the public health epidemic of MetS. One of the limitations of the present study is that there is no active control group in the study design. It is not known whether the establishment of a regular exercise habit and regular group gathering might contribute a portion of the beneficial effects to our examined outcome measures due to the change of the exercise habit and social life other than the yoga intervention and it remains to be further elucidated.
